# Association Between Androgen Deprivation Therapy Use and Diagnosis of Dementia in Men With Prostate Cancer

**DOI:** 10.1001/jamanetworkopen.2019.6562

**Published:** 2019-07-03

**Authors:** Ravishankar Jayadevappa, Sumedha Chhatre, S. Bruce Malkowicz, Ravi B. Parikh, Thomas Guzzo, Alan J. Wein

**Affiliations:** 1 Leonard Davis Institute of Health Economics, Perlman School of Medicine, University of Pennsylvania, Philadelphia; 2 Perlman School of Medicine, Division of Urology, Department of Surgery, University of Pennsylvania, Philadelphia; 3 Perlman School of Medicine, Department of Medicine, University of Pennsylvania, Philadelphia; 4 Perlman School of Medicine, Department of Psychiatry, University of Pennsylvania, Philadelphia

## Abstract

**Importance:**

The association between androgen deprivation therapy (ADT) exposure and dementia is uncertain.

**Objective:**

To analyze the association between ADT exposure and diagnosis of Alzheimer disease or dementia among elderly men with prostate cancer.

**Design, Setting, and Participants:**

This retrospective cohort study used data from the National Cancer Institute’s Surveillance, Epidemiology, and End Results–Medicare linked database. Participants were 154 089 elderly men newly diagnosed with prostate cancer between 1996 and 2003. The analyses were conducted between November 1, 2018, and December 31, 2018.

**Exposure:**

Androgen deprivation therapy.

**Main Outcomes and Measures:**

Patients receiving ADT within 2 years of prostate cancer diagnosis were identified. Survival analysis was used to determine the association between ADT exposure and diagnosis of Alzheimer disease or dementia in the follow-up period. Propensity score and instrumental variable approaches were used to minimize measured and unmeasured selection bias. The association by dose of ADT was also examined.

**Results:**

Of the 295 733 men diagnosed with prostate cancer between 1996 and 2003, 154 089 met the study criteria. Of these, 62 330 (mean [SD] age, 76.0 [6.0] years) received ADT within 2 years of prostate cancer diagnosis, and 91 759 (mean [SD] age, 74.3 [6.0] years) did not receive ADT. Mean (SD) follow-up was 8.3 (4.7) years. Exposure to ADT, compared with no ADT exposure, was associated with a diagnosis of Alzheimer disease (13.1% vs 9.4%; difference, 3.7%; 95% CI, 3.3%-3.9%; *P* < .001; hazard ratio [HR], 1.14; 95% CI, 1.10-1.18) and dementia (21.6% vs 15.8%; difference, 5.8%; 95% CI, 5.4%-6.2%; *P* < .001; HR, 1.20; 95% CI, 1.17-1.24). For 1 to 4 doses of ADT, the HR was 1.19 (95% CI, 1.15-1.24) for Alzheimer disease and 1.19 (95% CI, 1.15-1.23) for dementia. For 5 to 8 doses of ADT, the HR was 1.28 (95% CI, 1.22-1.35) for Alzheimer disease and 1.24 (95% CI, 1.19-1.29) for dementia. For more than 8 doses of ADT, the HR was 1.24 (95% CI, 1.16-1.34) for Alzheimer disease and 1.21 (95% CI, 1.15-1.28) for dementia. The number needed to harm was 18 patients (95% CI, 17-19 patients) and 10 patients (95% CI, 9.5-11 patients) for Alzheimer disease and dementia, respectively.

**Conclusions and Relevance:**

Among elderly patients with prostate cancer, ADT exposure was associated with subsequent diagnosis of Alzheimer disease or dementia over a follow-up period of at least 10 years.

## Key Points

**Question:**

Is androgen deprivation therapy exposure associated with dementia among elderly patients with prostate cancer?

**Findings:**

In this cohort study of 154 089 elderly men with prostate cancer, androgen deprivation therapy exposure was associated with subsequent diagnosis of Alzheimer disease or dementia over a follow-up period of at least 10 years.

**Meaning:**

Clinicians must carefully weigh the long-term risks and benefits of exposure to androgen deprivation therapy in patients with a prolonged life expectancy and stratify patients by dementia risk prior to androgen deprivation therapy initiation.

## Teaser

This cohort study uses data from the Surveillance, Epidemiology, and End Results (SEER)–Medicare database to investigate the association between androgen deprivation therapy and diagnosis of Alzheimer disease or dementia among elderly men with prostate cancer.

## Introduction

Prostate cancer is the most common nonskin cancer among men in the United States.^1^ Androgen deprivation therapy (ADT), alone or as part of multimodal therapy, reduces the likelihood of cancer progression and/or mortality in high-risk localized, locally advanced, recurrent, or metastatic prostate cancer.^2,3,4,5,6,7,8,9^ Despite these benefits, ADT may have long-term effects on bone, sexual, and cardiovascular health that influence prostate cancer–related quality of life, functional status, and health care utilization.^10,11,12,13,14,15,16,17,18,19^

The possible association between ADT exposure and cognitive dysfunction represents a growing concern. There are several purported mechanisms behind this association. Decreasing androgen levels may increase risk factors for Alzheimer disease and dementia, including loss of lean body mass, diabetes, cardiovascular disease, and depression.^11^ There may be a causative relationship between lower testosterone levels and impaired cognitive function, perhaps via impaired neuron growth and axonal regeneration or accumulation of abnormally folded β-amyloid protein.^10,12,13,14,19^ Studies using national samples have reported conflicting results regarding the diagnosis of Alzheimer disease or dementia among older patients with prostate cancer exposed to ADT.^17,18,20,21,22,23^ Limitations of these studies include inadequate adjustment for cancer stage, ADT dose, and duration; reliance on single-institution data; lack of generalizability to the US population; and measured and unmeasured bias associated with cohort studies. Clarifying the association between ADT and dementia could improve shared decision making around the risks and benefits of ADT in prostate cancer. The objective of this study was to investigate the association between exposure to ADT and subsequent diagnosis of Alzheimer disease or dementia among elderly men with prostate cancer. We hypothesized that, after adjusting for relevant covariates, ADT exposure is associated with an increased hazard of subsequent dementia.

## Methods

### Data Sources

The Surveillance, Epidemiology, and End Results (SEER)–Medicare linked database of the National Cancer Institute brings together Medicare administrative claims data and clinical tumor registry data for Medicare recipients who reside in the SEER region.^24^ The SEER program collects data on cancer incidence, treatment, and mortality from 18 SEER sites and encompasses 28% of the US population. Of persons diagnosed with cancer aged 65 years and older enrolled in SEER registries, 93% have been matched with Medicare enrollment records.

For our retrospective cohort study, we obtained data from a sample of men aged 66 years and older and diagnosed with localized or advanced prostate cancer between 1996 and 2003 from the SEER-Medicare database. This cohort was followed up until 2013; thus, each patient potentially had at least 10 years of follow-up after diagnosis of prostate cancer. Patients who were younger than 66 years at the time of diagnosis were excluded to ensure that the data file included sufficient claims for medical care prior to the diagnosis of prostate cancer to allow the adjustment for prediagnosis comorbidity. The 2-year period after diagnosis was considered the treatment phase, and the following years were considered the follow-up phase. Patients who received orchiectomy within 2 years of prostate cancer diagnosis were included. To analyze the association with dose, ADT was stratified according to the number of doses received within 2 years of prostate cancer diagnosis (1-4, 5-8, and >8 doses).^25^ The analyses were conducted between November 1, 2018, and December 31, 2018, and all data were deidentified. This report follows the Strengthening the Reporting of Observational Studies in Epidemiology (STROBE) reporting guideline. This study was approved by the University of Pennsylvania institutional review board. Requirements for informed consent were waived because the data were deidentified.

### Measurements

#### Diagnosis of Dementia and Alzheimer Disease

Diagnosis of dementia and Alzheimer disease were the key outcome variables in our analyses. Diagnostic codes included in Medicare inpatient, outpatient, and provider claims (*provider claims* is the term used by Medicare to indicate claims filed by health care professionals in the Medicare system) were used to identify patients with a diagnosis of dementia (*International Classification of Diseases, Ninth Revision, Clinical Modification* [*ICD-9-CM*] codes 290, 29420, 29411, 29282, 2912, and 29421) or Alzheimer disease (*ICD-9-CM* code 3310) after diagnosis of prostate cancer. We excluded patients with a preexisting diagnosis of Alzheimer disease or dementia.

#### Covariates

We obtained sociodemographic, disease severity, medical comorbidity, and prostate cancer treatment characteristics for use in adjusting our measures of association for potentially influential covariates. Age, race/ethnicity, socioeconomic stats (SES), and geographic location data were obtained from the SEER-Medicare Patient Entitlement and Diagnosis Summary File. Prostate cancer severity was assessed with information on prostate cancer grade and histology provided in SEER. Charlson Comorbidity Indexes were generated for each patient using the inpatient, outpatient, and provider claims in the 1-year period prior to the diagnosis of prostate cancer.^26^ In addition to treatment information from the Patient Entitlement and Diagnosis Summary File, procedure codes were used to identify prostate cancer treatments. Treatments were surgery, radiation therapy (external beam or brachytherapy), chemotherapy, ADT, or no treatment.

### Statistical Analysis

We used unpaired 2-tailed *t* tests, or χ^2^ tests, as appropriate, to test the significance of the differences between continuous and categorical variables. In all analyses, 2-sided *P* < .05 was considered statistically significant. We used 3 sequential models to analyze the association between ADT and diagnosis of Alzheimer disease (or dementia).^27,28,29^ The key independent variable was exposure to ADT. We operationalized exposure to ADT in 2 ways: as a binary variable and as intensity of use. Survival analysis was used to study the association between ADT and Alzheimer disease (or dementia). Model 1 estimated the unadjusted association of ADT with Alzheimer disease (or dementia). To minimize confounding by indication, we used propensity score analysis (model 2) and instrumental variable analysis (data not shown) to examine the relationship between ADT and subsequent Alzheimer disease (or dementia). For model 2, using logistic regression, we first estimated for each participant the probability (ie, the propensity) of receiving ADT based on age, race/ethnicity, geographic location, SES, marital status, and Charlson Comorbidity Index score.^28^ Next, in model 2, we modeled the associations between Alzheimer disease (or dementia) and ADT, weighted by the inverse propensity score, after adjusting for age, race/ethnicity, geographic location, SES, marital status, Charlson comorbidity score, stage, treatment, and year of diagnosis. To examine the effect of weighting, we compared the covariates before and after adjustment for propensity score.

We used an instrumental variable approach to address unmeasured bias, relying on an instrumental variable that is associated with the likelihood of receiving a type of treatment but is independent of diagnosis of Alzheimer disease or dementia.^30^ An appropriate instrument is one that is associated with the exposure (ADT treatment) but not with the outcome(s). There exists variation in treatment across regions, and therefore patients from these regions may be more likely to receive ADT treatment for prostate cancer. Therefore, for each hospital referral region in our study, we determined the proportion of patients who received ADT treatment. We categorized hospital referral regions as high- or low-treatment regions using the median as a cutoff and used this variable as an instrument. Our instrument satisfied 2 criteria for being a useful instrument. First, the Hausman test of endogeneity showed that an instrumental variable approach was necessary for our model. Second, our instrument fulfilled a rule of thumb in the literature that the weak instruments problem is a nonissue if the *F* statistic of the regression in the reduced form equation exceeds 10.

We also conducted 4 types of sensitivity analysis: (1) timing of ADT use—narrowing the primary treatment phase from 2 years to 6 months after diagnosis; (2) subgroups of comorbidity—those with no comorbidity, those with 1 to 2 comorbidities, and those with more than 2 comorbidities; (3) stage of cancer—localized vs advanced; and (4) other treatment groups. We used SAS statistical software version 9.4 (SAS Institute Inc) for analysis.

## Results

### Sample Characteristics

**Figure 1.**
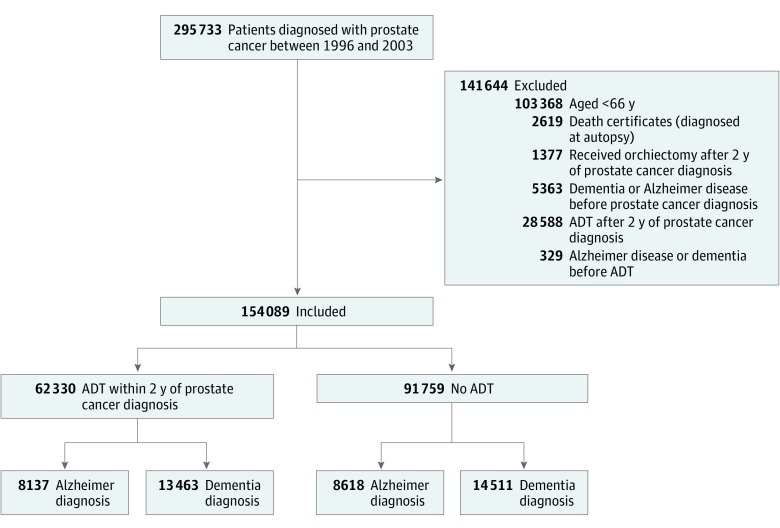
Process of Cohort Selection ADT indicates androgen deprivation therapy.

Of the 295 733 Medicare fee-for-service beneficiaries newly diagnosed with prostate cancer between 1996 and 2003, 154 089 met our study criteria (Figure 1). Of these, 62 330 received ADT within 2 years of prostate cancer diagnosis and 91 759 did not receive ADT by the end of the study (December 31, 2013). A list of diagnosis and procedure codes used is presented in eTable 1 in the Supplement. As shown in Table 1, those who received ADT were older at diagnosis of prostate cancer (mean [SD] age, 76.0 [6.0] years) compared with those who did not receive ADT (mean [SD] age, 74.3 [6.0] years). Men receiving ADT were more likely than men not receiving ADT to live in nonmetropolitan areas (16.7% vs 10.4%; difference, 6.3%; 95% CI, 5.9%-6.7%; *P* < .001), to be unmarried (33.7% vs 30.4%; difference, 3.3%; 95% CI, 2.9%-3.8%; *P* < .001), and to have lower SES (38.8% vs 33.4%; difference, 5.3%; 95% CI, 4.8%-5.8%; *P* < .001). Men receiving ADT were more likely to have at least 1 comorbidity (32.0% vs 17.4%; difference, 14.6%; 95% CI, 14.1%-15.1%; *P* < .001) and have more aggressive cancer than those not receiving ADT (34.3% vs 17.8%; difference, 16.5%; 95% CI, 16.1%-16.9%; *P* < .001). Those receiving ADT were less likely to have undergone radical prostatectomy compared with those not receiving ADT (18.9% vs 26.4%; difference, 7.3%; 95% CI, 6.9%-7.8%; *P* < .001). Mean (SD) follow-up was 8.3 (4.7) years.

**Table 1.** Demographic and Clinical Characteristics of Men Aged 66 Years and Older Diagnosed With Prostate Cancer Between 1996 and 2003, According to ADT Status

Variable	Unadjusted			Propensity Score–Adjusted^a^		
	No ADT, No. (%) (n = 91 759)	ADT, No. (%) (n = 62 330)	*P* Value	No ADT, No. (%) (n = 93 238)^b^	ADT, No. (%) (n = 59 480)^b^	*P* Value
Age at diagnosis, mean (SD), y	74.3 (6.0)	76.0 (6.0)	<.001	75.2 (6.4)	75.2 (5.9)	.29
Ethnicity						
White	69 053 (75.3)	48 480 (77.8)	<.001	70 912 (78.1)	45 656 (76.7)	.01
African American	10 286 (11.2)	6385 (10.2)		10 361 (11.1)	6492 (10.9)	
Hispanic	5814 (6.3)	3307 (5.3)		5625 (6.8)	3958 (6.7)	
Other	6606 (7.2)	4158 (6.7)		6341 (6.8)	3958 (6.7)	
Marital status						
Married	63 907 (69.7)	41 324 (66.3)	<.001	63 127 (67.7)	40 889 (68.8)	<.001
Single, separated, or divorced	27 852 (30.4)	21 006 (33.7)		30 111 (32.3)	18 590 (31.3)	
Geographic area						
Metropolitan	82 228 (89.6)	51 919 (83.3)	.001	81 121 (87.0)	51 008 (85.8)	<.001
Urban	8447 (9.2)	9185 (14.7)		10 772 (11.6)	7410 (12.5)	
Rural	1084 (1.2)	1226(1.9)		1345 (1.4)	1063 (1.8)	
Charlson Comorbidity Index						
0	75 763 (82.6)	42 344 (68.0)	<.001	70 713 (75.8)	44 056 (74.1)	<.001
1-2	13 788 (15.0)	17 529 (28.1)		19 753 (26.8)	13 213 (22.2)	
>3	2208 (2.4)	2457 (3.9)		2772 (2.9)	2211 (3.7)	
Socioeconomic status						
Low	30 019 (33.5)	23 536 (38.8)	<.001	32 398 (34.8)	21 523 (36.2)	<.001
Medium	22 471 (25.1)	14 741 (24.8)		24 953 (26.8)	16 075 (27.0)	
High	37 200 (41.5)	22 445 (37.0)		35 887 (38.5)	21 881 (36.8)	
Year of diagnosis						
1996	7769 (8.4)	3839 (6.2)	<.001	7959 (8.5)	5381 (9.1)	<.001
1997	7855 (8.6)	4243 (6.8)		7782 (8.4)	4880 (8.2)	
1998	7747 (8.4)	4380 (7.0)		7675 (8.2)	5049 (8.5)	
1999	8006 (8.7)	4964 (7.9)		7904 (8.5)	4916 (8.3)	
2000	15 588 (17.0)	11 073 (17.8)		15 582 (16.7)	10 253 (17.6)	
2001	15 576 (16.9)	11 588 (18.6)		15 530 (16.7)	10 465 (17.6)	
2002	15 473 (16.9)	11 725 (18.8)		15 784 (16.9)	10 135 (17.0)	
2003	13 815 (15.1)	10 518 (16.9)		15 022 (16.1)	8401 (14.1)	
Cancer grade						
Well differentiated	5644 (6.2)	1953 (3.1)	<.001	4534 (4.9)	2847 (4.8)	<.001
Moderately differentiated	62 265 (67.9)	34 903 (56.0)		57 270 (61.4)	37 495 (63.0)	
Poorly differentiated or undifferentiated	16 310 (17.8)	21 376 (34.3)		23 629 (25.3)	148 993 (25.1)	
Unknown	7540 (8.2)	4098 (6.6)		7805 (8.4)	4239 (7.1)	
Treatment						
Surgery (monotherapy or multimodal therapy)	24 188 (26.4)	11 836 (18.9)	<.001	21 279 (22.8)	14 089 (23.6)	<.001
Radiation (monotherapy or multimodal therapy)	39 353 (43.0)	42 903 (68.8)		50 032 (53.7)	33 410 (56.2)	
Chemotherapy (alone)	243 (0.3)	7327 (11.8)		5111 (5.5)	3061 (5.2)	
No treatment	27 975 (30.5)	264 (0.4)		16 815 (18.0)	8919 (15.0)	
Diagnosis in the 2-y period after prostate cancer diagnosis						
Alzheimer disease	8618 (9.4)	8137 (13.1)	<.001	9073 (9.7)	6406 (10.8)	<.001
Dementia	14 511 (15.8)	13 463 (21.6)	<.001	15 602 (16.7)	10 495 (17.6)	<.001

Abbreviation: ADT, androgen deprivation therapy.

^a^ Adjusted for age at diagnosis, race/ethnicity, geographic area, marital status, comorbidity score, cancer stage, and socioeconomic status.

^b^ Numbers are synthetic values derived from weights.

### Diagnosis of Alzheimer Disease

**Figure 2.**
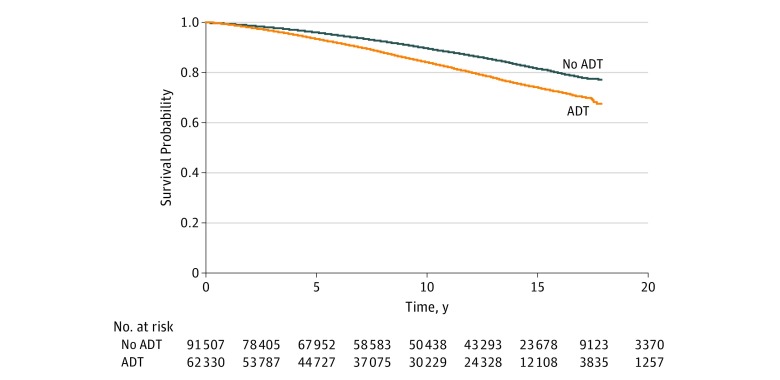
Survival Curve for Alzheimer Disease Patients exposed to androgen deprivation therapy (ADT) had a higher hazard of diagnosis of Alzheimer disease compared with those not exposed to ADT.

As reported in Table 1, patients with prostate cancer treated with ADT were more likely to be diagnosed with Alzheimer disease compared with those not treated with ADT (13.1% vs 9.4%; difference, 3.7%; 95% CI, 3.3%-3.9%; *P* < .001). Survival analyses evaluated the hazard of Alzheimer disease diagnosis and ADT exposure (Table 2). Overall, patients with prostate cancer receiving ADT had a higher hazard of Alzheimer disease (hazard ratio [HR] for propensity score approach, 1.14; 95% CI, 1.10-1.18). The instrumental variable model yielded comparable results (data not shown). The experience with Alzheimer disease diagnosis differed between the ADT and non-ADT groups (log-rank *P* < .001) (Figure 2).

**Table 2.** Association Between ADT and Diagnosis of Alzheimer Disease or Dementia

Model	Hazard Ratio (95% CI)	
	Alzheimer Disease	Dementia
**Association Between ADT and Diagnosis of Alzheimer Disease or Dementia**		
Unadjusted	1.56 (1.51-1.60)	1.61 (1.57-1.65)
Propensity score–adjusted^a^^,^^b^	1.14 (1.10-1.18)	1.20 (1.17-1.24)
**Association Between ADT Dose and Diagnosis of Alzheimer Disease or Dementia**^c^		
Unadjusted		
1-4 ADT doses	1.41 (1.36-1.46)	1.40 (1.37-1.44)
5-8 ADT doses	2.03 (1.94-2.12)	1.99 (1.93-2.07)
>8 ADT doses	1.94 (1.82-2.08)	1.96 (1.86-2.08)
No ADT	1 [Reference]	1 [Reference]
Propensity score–adjusted^a^^,^^b^		
1-4 ADT doses	1.19 (1.15-1.24)	1.19 (1.15-1.23)
5-8 ADT doses	1.28 (1.22-1.35)	1.24 (1.19-1.29)
>8 ADT doses	1.24 (1.16-1.34)	1.21 (1.15-1.28)
No ADT	1 [Reference]	1 [Reference]

Abbreviation: ADT, androgen deprivation therapy.

^a^ Adjusted for age at diagnosis, race/ethnicity, geographic area, marital status, comorbidity score, cancer stage, and socioeconomic status.

^b^ Inverse probability of treatment weighting.

^c^ *P* values for trend are <.001.

### Diagnosis of Dementia

**Figure 3.**
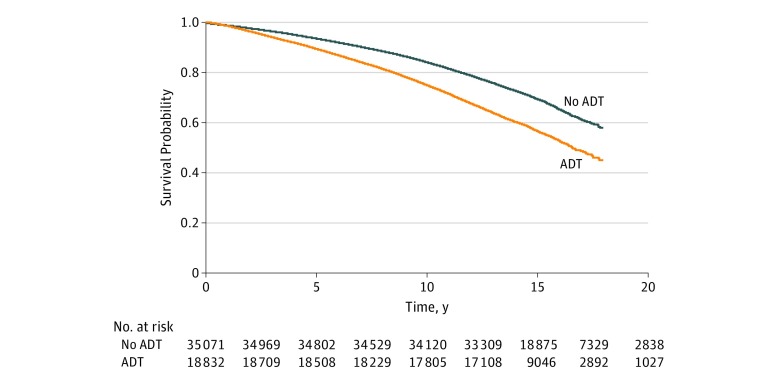
Survival Curve for Dementia A significantly higher hazard of diagnosis of dementia was observed for patients treated with androgen deprivation therapy (ADT) vs those not treated with ADT.

Similar to Alzheimer disease, patients with prostate cancer exposed to ADT experienced a higher likelihood of dementia diagnosis compared with patients who were not exposed to ADT (21.6% vs 15.8%; difference, 5.8%; 95% CI, 5.4%-6.2%; *P* < .001), as reported in Table 1. Survival analyses evaluated the association between exposure to ADT and hazard of diagnosis of dementia after adjusting for inversely weighted propensity score (Table 2). Overall, patients receiving ADT had a higher hazard of being diagnosed with dementia (HR for propensity score approach, 1.20; 95% CI, 1.17-1.24). Comparable results were observed with instrumental variable analysis (data not shown). The experience with dementia differed between the ADT group and the non-ADT group (log-rank *P* < .001) (Figure 3).

### Dose Effect and Numbers Needed to Harm

As shown in Table 2, the association between prolonged ADT use (5-8 doses) and diagnosis of Alzheimer disease (HR, 1.28; 95% CI, 1.22-1.35) was consistent with results for overall exposure to ADT. Similarly, prolonged ADT was associated with an increased hazard of dementia (HR, 1.24; 95% CI, 1.19-1.29). For 1 to 4 doses of ADT, the HR was 1.19 (95% CI, 1.15-1.24) for Alzheimer disease and 1.19 (95% CI, 1.15-1.23) for dementia. For 5 to 8 doses of ADT, the HR was 1.28 (95% CI, 1.22-1.35) for Alzheimer disease and 1.24 (95% CI, 1.19-1.29) for dementia. For more than 8 doses of ADT, the HR was 1.24 (95% CI, 1.16-1.34) for Alzheimer disease and 1.21 (95% CI, 1.15-1.28) for dementia. All *P* values for trend were less than .001. The number needed to harm was 18 patients (95% CI, 17-19 patients) for Alzheimer disease and 10 patients (95% CI, 9.5-11 patients) for dementia.

### Sensitivity Analyses

We conducted several types of sensitivity analyses to assess the robustness of the association between ADT and diagnosis of Alzheimer disease (or dementia). First we analyzed the association for 3 subgroups of comorbidity (0 comorbidities, 1-2 comorbidities, and >2 comorbidities) (eTable 2 in the Supplement). Only the group with 0 comorbidities showed an association between exposure to ADT and diagnosis of Alzheimer disease (HR, 1.22; 95% CI, 1.17-1.27) or dementia (HR, 1.28; 95% CI, 1.24-1.32). Next, we analyzed subgroups of treatment combination. Among the surgery group, ADT use had a lower hazard of diagnosis of Alzheimer disease (HR, 0.92; 95% CI, 0.85-0.98) and a higher hazard of dementia (HR, 1.16; 95% CI, 1.10-1.23) (eTable 3 in the Supplement). For the radiation and chemotherapy groups, the association between exposure to ADT and diagnosis of Alzheimer disease (or dementia) was higher than that observed in Table 2 (eTable 4 in the Supplement). Additionally, for the localized and advanced stage subgroups, the association between ADT and Alzheimer disease (or dementia) was comparable to that observed in Table 2. Varying primary treatment period for ADT of 6 months showed similar results (data not reported).

## Discussion

Using SEER-Medicare linked data, we demonstrated that exposure to ADT was associated with an increased hazard of subsequent Alzheimer disease or dementia among patients with prostate cancer over a mean follow-up of 8.3 years after prostate cancer diagnosis. This association continued with different treatment groups. Additionally, we observed a dose-response relationship: patients who received more than 8 doses of ADT were at a significantly higher hazard of diagnosis of both dementia and Alzheimer disease than those receiving fewer doses ADT.

To our knowledge, this is the largest study to date examining the association between exposure to ADT and subsequent dementia in a US cohort of elderly patients with prostate cancer. There has been conflicting evidence in studies examining association between ADT use and dementia diagnosis.^10,25,31,32,33,34,35,36^ Prior observational studies that did not observe an association between ADT and dementia may have been limited by varying inclusion criteria, inability to account for duration of ADT, and short follow-up time. Our accounting for these factors may explain the discrepancies in findings between these studies and ours.^10,11,35,36,37^ A meta-analysis^12^ revealed an association between ADT exposure and poorer performance on testing of several cognitive domains; however, the studies that were included were limited by heterogeneity in study population. Strengths of our study include its large sample of elderly US men, long follow-up time, establishment of a dose-response association, adjustment for unmeasured and measured confounding by indication, and consistent association across treatment, comorbidity, and cancer grade subgroups.

While our study suggests an association between ADT and subsequent dementia diagnosis, we were unable to further investigate possible biological mechanisms of this association. It is important to note that dementia may have a latency period of 1 decade or more prior to cognitive manifestations,^38^ with some cerebrospinal fluid, serum, and neuroimaging biomarkers present many years before diagnosis.^39^ Hence, it is possible that ADT has a modifying or augmenting, rather than de novo, effect on development of dementia. Further work could be done to characterize individuals undergoing ADT who are at high risk of developing earlier dementia.

### Limitations

We note some limitations to our study. Our sample was limited to male fee-for-service Medicare enrollees aged 66 years or older who lived in a SEER region and were not enrolled in a health maintenance organization; thus, the results may not be generalizable to other populations. Furthermore, while the age and sex distribution for persons aged 66 years and older is comparable with that of older adults in the United States, SEER regions have a higher proportion of nonwhite individuals. Mortality rates derived from SEER data may not be representative of national data on cancer mortality rates.^24^ While the threshold for Alzheimer disease or dementia diagnosis may vary from physician to physician,^40^ studies of claims data for Alzheimer disease or dementia have shown generally good agreement between physicians and practices.^10^ Nevertheless, Alzheimer disease or dementia may be a challenge to diagnose in older men, and physicians may vary widely in recognition of and inclination to code for Alzheimer disease or dementia.^40^ Our estimates of the association of ADT with diagnosis of Alzheimer disease or dementia may be conservative because men with Alzheimer disease or dementia may be misclassified into the comparison group in the absence of a diagnostic code for dementia.

Future research should attempt to elucidate a possible biological mechanism between exposure to ADT and development of dementia and study this association prospectively. It is crucial to establish whether this association is mediated by long-term androgen suppression, especially as dual androgen blockade with second-generation antiandrogens moves earlier in the treatment course of prostate cancer.^41,42^ It is unclear whether prolonged exposure to dual androgen blockade increases the risk of subsequent dementia.

## Conclusions

In summary, our population-based study spanning 10 years or more following the diagnosis of prostate cancer shows that exposure to ADT was associated with increased hazard of both Alzheimer disease and dementia among elderly fee-for-service Medicare beneficiaries with prostate cancer. The list of effective ADT agents has recently grown with the addition of androgen synthesis inhibitors and second-generation antiandrogens. Furthermore, data are accumulating for the use of such agents earlier in the course of disease progression. Our results suggest that clinicians need to carefully weigh the long-term risks and benefits of exposure to ADT in patients with a prolonged life expectancy and stratify patients based on dementia risk prior to ADT initiation.

## Notes

Supplement. **eTable 1.** Diagnosis and Procedure Codes **eTable 2.** Association Between ADT and Diagnosis of Alzheimer’s or Dementia (Comorbidity Score Subgroups) **eTable 3.** Association Between ADT and Diagnosis of Alzheimer’s or Dementia (Treatment Type Subgroups) **eTable 4.** Association Between ADT and Diagnosis of Alzheimer’s or Dementia (Cancer Stage)
