# Supplementary material for: Association Between Androgen Deprivation Therapy Use and Diagnosis of Dementia in Men With Prostate Cancer
Source: JAMA Netw Open. 2019 Jul 3;2(7):e196562. doi: 10.1001/jamanetworkopen.2019.6562 (PMC6613289; doi:10.1001/jamanetworkopen.2019.6562)
Supplement: Supplement. — eTable 1. Diagnosis and Procedure Codes eTable 2. Association Between ADT and Diagnosis of Alzheimer’s or Dementia (Comorbidity Score Subgroups) eTable 3. Association Between ADT and Diagnosis of Alzheimer’s or Dementia (Treatment Type Subgroups) eTable 4. Association Between ADT and Diagnosis of Alzheimer’s or Dementia (Cancer Stage) [file jamanetwopen-2-e196562-s001.pdf]

Supplementary Online Content

Jayadevappa R, Chhatre S, Malkowicz SB, Parikh RB, Guzzo T, Wein AJ. Association between androgen deprivation therapy use and diagnosis of dementia in men with prostate cancer. *JAMA Netw Open*. 2019;2(7):e196562. doi:10.1001/jamanetworkopen.2019.6562

eTable 1. Diagnosis and Procedure Codes

eTable 2. Association Between ADT and Diagnosis of Alzheimer's or Dementia (Comorbidity Score Subgroups)

eTable 3. Association Between ADT and Diagnosis of Alzheimer's or Dementia (Treatment Type Subgroups)

eTable 4. Association Between ADT and Diagnosis of Alzheimer's or Dementia (Cancer Stage)

This supplementary material has been provided by the authors to give readers additional information about their work.

© 2019 Jayadevappa R et al. *JAMA Network Open.*

eTable1: Diagnosis and procedure codes

	ICD 9 dxs code	ICD 9 procedure code	HCPCS/CPT code
Diagnosis			
Prostate cancer dx	185		
Alzheimer	3310		
Dementia	290, 29420 29411, 29410, 29282, 2912, 29421		
Treatment			
Hormone (androgen deprivation therapy or ADT)	V07.59		C9216, C9430, G0356, G9132, J0128, J1050, J1051, J1950, J3315, J9165, J9202, J9217-J9219, S0165, S0175, S9560
Orchiectomy		62.3, 62.4, 62.41, 62.42	54520, 54521, 54522, 54530, 54535,
Surgery<br>Radical prostatectomy<br>Laparoscopic (radical prostatectomy,<br>Robotic-assisted laparoscopic radical prostatectomy)		17.42, 40.1, 40.2, 40.3, 40.5, 40.53, 40.59, 60.21, 60.29, 60.2–60.6, 60.51-60.59, 60.61, 60.62, 60.69	54690, 55810, 55812, 55815, 55821, 55831, 55840, 55842, 55845, 55866, 55899, S2900
Cryosurgery (*cryotherapy/ cryoablation*)			55873
Radiation : EBRT	V58.0, V66.1, V67.1	60.99, 92.2, 92.20, 92.21-92.29, 92.3, 92.30-92.39, 92.4, 92.41	0073T, 0082T, 0182T, 0197T, 55860, 55862, 55865, 55875, 55876, 61793, 76872, 76873, 76965, 77261–79999, C1715, C1717, C2638-C2641, C2698, C2699, G0173, G0251, G0256, G0261, G0339, G0340, G0458, G6003, G6005, G6006, G6015, Q3001, S8049; Revenue center codes 0330 or 0333, 0339 or 0342
Radiation: brachytherapy			0182T, 77750-77799, C1715, C1716, C1717, C1719,C2616, C2634-C2645, C2698, C2699, G0458, Q3001
Chemo tx	V58.1<br>V66.2, V67.2	99.25	95990, 95991, 96400-96549, 96530, J0640, J2405, J8520 - J9999 K0415, K0416, Q0083-Q0085, Q0179, S0177, S0181 Revenue center codes 0331, 0332, or 0335; For 2005 only, use these G0355-G0363, G9021-G9032

© 2019 Jayadevappa R et al. *JAMA Network Open.*

eTable2: Association between ADT and diagnosis of Alzheimer’s or dementia (comorbidity score subgroups). Hazard ratio represent the comparison between patients with ADT (within 24 months of diagnosis) and those without ADT - sub groups of comorbidity score

**Model**	**Comorbidity score**					
	**Zero**		**1-2**		**>2**	
	**Alzheimer’s HR (CI)**	**Dementia HR (CI)**	**Alzheimer’s HR (CI)**	**Dementia HR (CI)**	**Alzheimer’s HR (CI)**	**Dementia HR (C**
**Unadjusted**						
	1.61 (1.55, 1.67)	1.65 (1.60, 1.69)	1.09 (1.02, 1.16)	1.14 (1.09, 1.19)	0.92 (0.77, 1.09)	0.91 (0.80, 1.03)
**Propensity score adjusted * (IPTW)****						
	1.22 1.17, 1.27)	1.28 (1.24,1.32)	0.93 (0.87, 1.00)	1.02 (0.96, 1.08)	0.88 (071, 1.09)	0.98 (0.83, 1.15)

* Adjusted for age at diagnosis, race and ethnicity, geographic area, marital status, caner stage, treatment type and socio-economic status
** Inverse probability of treatment weighting

© 2019 Jayadevappa R et al. *JAMA Network Open.*

eTable3: Association between ADT and diagnosis of Alzheimer's or dementia (treatment type subgroups). Hazard ratio represent the comparison between patients with ADT (within 24 months of diagnosis) and those without ADT - sub groups of treatment type

Model	Treatment type					
	Surgery[$]		Radiation[$$]		Chemotherapy[$$$]	
	Alzheimer's HR(CI)	Dementia HR (CI)	Alzheimer's HR(CI)	Dementia HR (CI)	Alzheimer's HR(CI)	Dementia HR (CI)
**Unadjusted**						
	1.31 (1.22, 1.41)	1.36 (1.28, 1.43)	1.54 (1.48, 1.60)	1.57 (1.52, 1.62)	2.02 (1.34, 3.05)	1.83 (1.36, 2.46)
**Propensity score adjusted * (IPTW)****						
	0.92 (0.85, 0.98)	1.16 (1.10, 1.23)	1.22 (1.17, 1.27)	1.21 (1.67, 1.24)	1.44 (0.96, 2.16)	1.24 (0.93, 1.65)

$ sub group of surgery (surgery alone or with radiation/chemo/ADT)
$$ sub group of radiation (radiation alone or with chemo/ADT)
$$$ sub group of chemotherapy (chemotherapy alone or with ADT)
* Adjusted for age at diagnosis, race and ethnicity, geographic area, marital status, comorbidity score, caner stage, and socio-economic status;
** Inverse probability of treatment weighting

© 2019 Jayadevappa R et al. *JAMA Network Open.*

eTable4: Association between ADT and diagnosis of Alzheimer's or dementia (cancer stage). Hazard ratio represent the comparison between patients with ADT (within 24 months of diagnosis) and those without ADT – localized vs. advanced stage

**Model**	**Cancer stage**			
	**Localized**		**Advanced**	
	**Alzheimer's HR (CI)**	**Dementia HR (CI)**	**Alzheimer's HR (CI)**	**Dementia HR (CI)**
**Unadjusted**				
	1.58 (1.52, 1.63)	1.62 (1.58, 1.67)	1.45 (1.35, 1.54)	1.51 (1.44, 1.59)
**Propensity score adjusted * (IPTW)****				
	1.15 (1.10, 1.20)	1.22 (1.19, 1.26)	1.08 (1.01, 1.16)	1.15 (1.09, 1.22)

* Adjusted for age at diagnosis, race and ethnicity, geographic area, marital status, comorbidity score, treatment type and socio-economic status;
** Inverse probability of treatment weighting

© 2019 Jayadevappa R et al. *JAMA Network Open.*
